# Distance Traveled by Colorectal Liver Metastasis Patients to the Nearest Cancer Hospital To Receive Liver Metastasectomy

**DOI:** 10.7759/cureus.82250

**Published:** 2025-04-14

**Authors:** Anne Pannekoek, Mengyuan Ruan, Mary Brindle, Quoc-Dien Trinh, George Molina

**Affiliations:** 1 Division of Surgical Oncology, Department of Surgery, Brigham and Women's Hospital, Harvard Medical School, Boston, USA; 2 Center for Surgery and Public Health, Brigham and Women's Hospital, Harvard Medical School, Boston, USA; 3 Ariadne Labs, Brigham and Women's Hospital, Harvard T.H. Chan School of Public Health, Boston, USA; 4 Urology, University of Pittsburgh Medical Center, Pittsburgh, USA

**Keywords:** colorectal liver metastasis, distance from cancer center, liver metastasectomy, primary colorectal cancer, unequal access to health care

## Abstract

Background

Liver metastasectomy for colorectal liver metastasis (CRLM) has been found to be associated with higher overall survival in select patients. However, this aggressive surgical treatment option is underutilized. At the county level, there is variation in undergoing surgery for CRLM that is associated with county-level poverty rates. However, in contrast, county-level variation in odds of undergoing surgery for stage I colorectal cancer (CRC) was not associated with county-level poverty rates. The objective of this study was to evaluate the impact of distance traveled as a barrier to surgery for CRLM compared to its impact on surgery for stage I CRC.

Methods

We previously used the Surveillance, Epidemiology, and End Results Research Plus (SEER) database to perform an ecological, cross-sectional, and county-level analysis of the county-level proportion of patients with CRLM diagnosed between January 1, 2010, and December 31, 2018, and as a comparator group, we included county-level proportion of patients with stage I CRC. In this study, we mapped out the variation in receiving surgery for CRLM or for stage I CRC that exists in states that contribute data to SEER. We also evaluated the correlation between the driving distance in miles from the center of a county to the closest National Cancer Institute (NCI) designated cancer center or Commission on Cancer (CoC) accredited cancer program and proportion of patients with CRLM and stage I CRC who received surgery at the county level.

Results

A total of 191 counties were included, and these included counties in which residing patients had access via land travel to an NCI-designated cancer center or CoC-accredited cancer program. Counties that had high rates of surgery for CRLM had the shortest distance to the nearest NCI-designated cancer center (63.1 and 46.5 miles, respectively). The distance to the nearest NCI-designated cancer center significantly differed between counties that were discordant and favored CRLM surgery and counties that were concordant and low for both CRC and CRLM surgery (p=0.02). The median distance to the nearest NCI-designated cancer center ranged from 46.5 miles to 114.5 miles among these four groups of categorized counties. Including the 25^th^ and 75^th^ percentiles, these traveling distances ranged from 25.8 miles to 218.3 miles.

Conclusion

Counties with high rates of surgery for CRLM did have the shortest distances to the nearest NCI-designated cancer center. Although median traveling distances to the nearest NCI-designated cancer center were potentially prohibitive, they did not fully explain differences in county-level surgical rates.

## Introduction

In highly selected patients, liver metastasectomy for colorectal liver metastasis (CRLM) is associated with higher overall survival [1]. However, liver metastasectomy for CRLM is underutilized, likely due to barriers that are multifactorial (e.g., patient, hospital, and geographic factors) [2-6]. Patients visiting hospitals with greater experience (i.e., hospitals with greater hospital-level and/or surgeon-level volume) and expertise (i.e., presence of specialized hepatobiliary surgeons) are more likely to receive liver surgery for CRLM [5,7-9]. We had previously found that patients with CRLM residing in counties with a higher proportion of their residents living below the federal poverty level were less likely to undergo a liver metastasectomy, but the same finding was not found when evaluating odds of undergoing surgery for stage I colorectal cancer (CRC) [10]. This study also demonstrated that there were concordant and discordant counties according to whether patients received or did not receive surgery for CRLM or stage I CRC. An underlying factor that might explain these findings is the distance to cancer hospitals since longer travel time to cancer care might be a financial, logistical, or psychological barrier to timely and appropriate cancer care.

To further explore these findings, we evaluated whether a longer distance to a National Cancer Institute (NCI)-designated cancer center or Commission on Cancer (CoC)-accredited cancer program was associated with concordant versus discordant receipt of surgery for CRLM and stage I CRC at the county level. Based on previous observations, we hypothesized that the impact of distance traveled to an NCI-designated cancer center or CoC-accredited cancer program would be the same for patients undergoing surgical interventions for CRLM or stage I CRC.

## Materials and methods

The Surveillance, Epidemiology, and End Results Research Plus (SEER) database was used in this ecological, cross-sectional, and county-level analysis of the county-level proportion of patients with CRLM diagnosed between January 1, 2010, and December 31, 2018, which has been described previously [10]. Patients with CRLM were included if they had their primary colorectal cancer resected and did not have extrahepatic disease. CRLM patients were excluded if they had missing data for any of the inclusion criteria. As a comparator group, the county-level proportion of patients with stage I CRC was included, and these included patients with stage I CRC who were also diagnosed between January 1, 2010 and December 31, 2018. In order to maintain patient privacy and per SEER data use agreement, counties with fewer than 10 patients in either group (i.e., CRLM or stage I CRC) were excluded from the analysis. Patients with stage I CRC with missing data for any of the inclusion criteria were excluded.

All counties with data that were reported in our previous publication were mapped according to poverty and resection rates using a tertile heatmap. This was performed to visually compare how surgical resection rates for CRLM and for stage I CRC differed according to poverty rates for the same county. Not all patients with CRLM will be candidates for liver metastasectomy. The most important criteria in determining whether a patient with CRLM is eligible for liver metastasectomy is whether the burden of liver disease is reseactable with a future liver remnant that is at least greater than 20% in patients without cirrhosis and who have not received chemotherapy and at least 30% in patients without cirrhosis and who have received chemotherapy. Another important criterion is lack of extrahepatic disease, with the exception of resectable lung metastasis. In contrast, most patients, if not all, with stage I CRC should receive surgery. Therefore, when categorizing county-level rates into tertiles, how each tertile was defined differed for CRLM and stage I CRC. Each tertile for CRLM and stage I CRC included an equal proportion of counties based on their rates of surgical resection for CRLM and stage I CRC, respectively. Our goal was to visually assess if relative low, medium, and high rates of surgery for CRLM and stage I CRC, respectively, followed the same pattern of relationship when mapped against county-level rates of poverty.

The proportion of patients at the county-level with CRLM and CRC undergoing surgery was categorized into four groups based on high and low surgical median volume of operations performed for CRLM and CRC, respectively. Using these four groups of counties, the nearest NCI-designated cancer centers were identified using the NCI Find a Cancer Center website [11]. The nearest CoC-accredited cancer programs were identified using the American College of Surgeons Hospitals and Facilities finder [12]. The distance between the center of each county and the location of the nearest NCI-designated cancer center or CoC-accredited cancer program was calculated using Google Maps. For some counties, the nearest NCI-designated cancer center or CoC-accredited cancer program was in a different state.

Statistical analysis

We calculated the median driving distance in miles between the center of a patient's county of residence with corresponding inter-quartile ranges (Q1-Q3) to the closest NCI-designated cancer center or CoC-accredited cancer program for each group of counties according to the proportion of patients with CRLM and CRC who received surgery at the county level.

The Kruskal-Wallis test was used to compare the distribution of counties according to the distance to the closest NCI-designated cancer center and CoC-accredited cancer program among the four groups. A sequentially rejective multiple test procedure according to Holm was used to adjust for multiple comparisons [13].

Surveillance Research Program SEER*Stat software, version 8.3.9.2 (National Cancer Institute), was used to extract the data. All analyses were performed using R statistical software (v4.1.0: R Core Team 2021, R Foundation for Statistical Computing, Vienna, Austria). P-value less than <0.05 was considered statistically significant.

## Results

**Figure 1:**
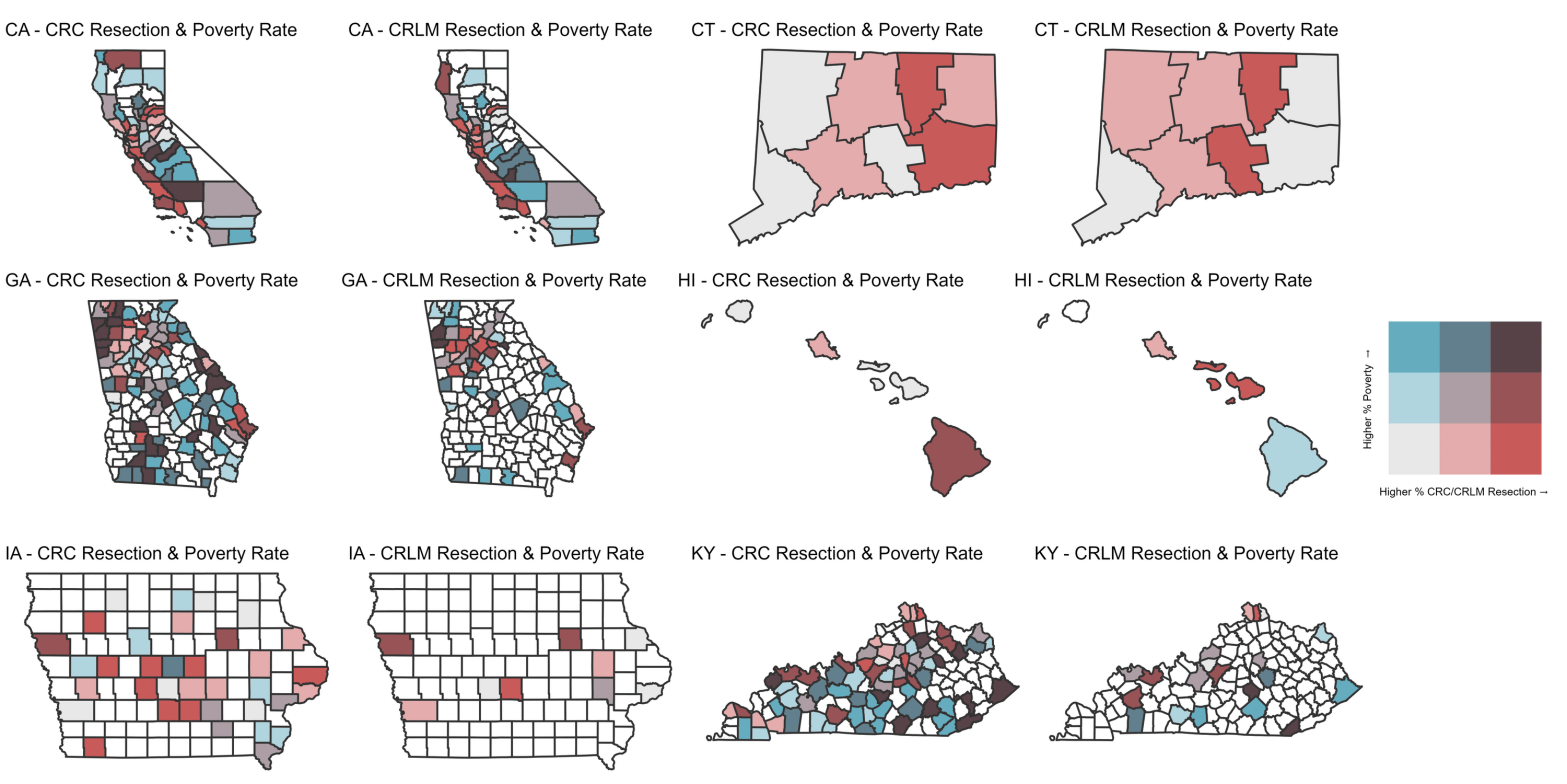
Bivariate state-county heatmaps showing relationship between rising county-level poverty rates and rising rates of surgery for colorectal liver metastasis (CRLM) and stage I colorectal cancer (CRC) for California, Connecticut, Georgia, Hawaii, Iowa, and Kentucky. CA: California, CT: Connecticut, GA: Georgia, HI: Hawaii, IA: Iowa, KY: Kentucky

**Figure 2:**
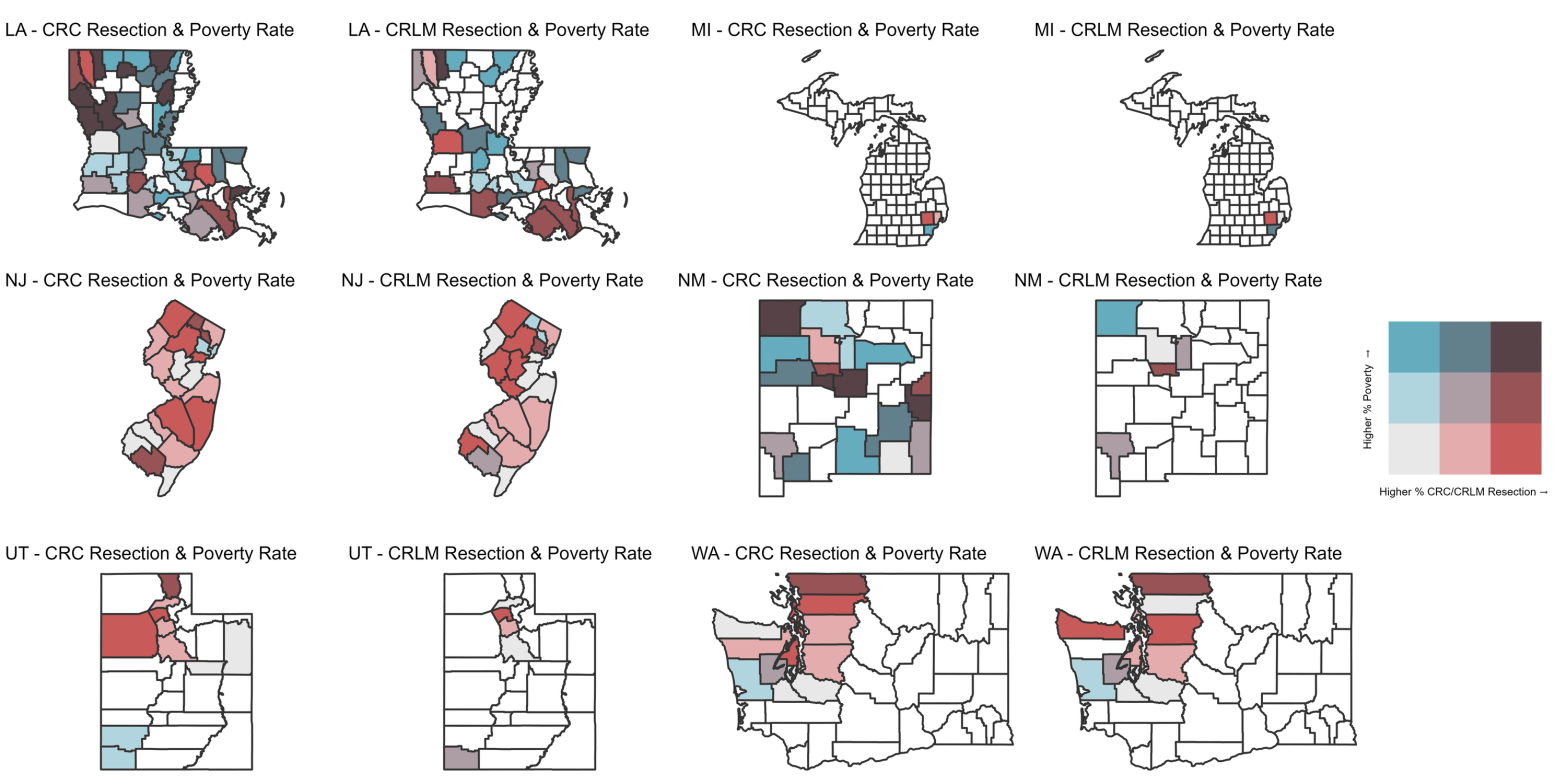
Bivariate state-county heatmaps showing relationship between rising county-level poverty rates and rising rates of surgery for colorectal liver metastasis (CRLM) and stage I colorectal cancer (CRC) for Louisiana, Michigan, New Jersey, New Mexico, Utah, and Washington. LA: Louisiana, MI: Michigan, NJ: New Jersey, NM: New Mexico, UT: Utah, WA: Washington

Using all 194 counties with available data on rates of surgery (surgery for CRLM and surgery for stage I CRC) and data for county-level poverty, state heatmaps were created to compare how the relationship between county-level poverty rates and rates of surgery differed for CRLM and stage I CRC (Figure 1, Figure 2). 

Many counties that had residents who received surgery for stage I CRC in California, Georgia, Iowa, Kentucky, Louisiana, and New Mexico, did not have residents who received surgery for CRLM. Additionally, rates of surgery categorized into tertiles, which differed for each state and cancer, differed according to county-level poverty rates (Table 1).

**Table 1:** Rates of surgery categorized into poverty rate tertiles, for each state and cancer Data is represented as rates (%) of surgery categorized into poverty rate tertiles (T1: Tertile 1, poverty rate < 13.7%; T2: Tertile 2, poverty rate 13.7% to 19.7%; T3: Tertile 3, poverty rate > 19.7%). CRLM: colorectal liver metastasis, CRC: colorectal cancer, CA: California, CT: Connecticut, GA: Georgia, HI: Hawaii, IA: Iowa, KY: Kentucky, LA: Louisiana, MI: Michigan, NJ: New Jersey, NM: New Mexico, UT: Utah, WA: Washington

State	Cancer	T1	T2	T3
CA	CRC Resection Rate	<71.4%	71.4% - 76.1%	>76.1%
CA	CRLM Resection rate	<18.5%	18.5% - 23.9%	>23.9%
CT	CRC Resection Rate	<72.7%	72.7% - 77.8%	>77.8%
CT	CRLM Resection rate	<26.5%	26.5% - 26.8%	>26.8%
GA	CRC Resection Rate	<75.8%	75.8% - 80.5%	>80.5%
GA	CRLM Resection rate	<23.7%	23.7% - 30.4%	>30.4%
HI	CRC Resection Rate	<76.1%	76.10%	>76.1%
HI	CRLM Resection rate	<19.7%	19.70%	>19.7%
IA	CRC Resection Rate	<77.4%	77.4% - 84.7%	>84.7%
IA	CRLM Resection rate	<14.3%	14.3% - 25.0%	>25.0%
KY	CRC Resection Rate	<73.9%	73.9% - 80.6%	>80.6%
KY	CRLM Resection rate	<21.7%	21.7% - 31.4%	>31.4%
LA	CRC Resection Rate	<69.0%	69.0% - 72.7%	>72.7%
LA	CRLM Resection rate	<18.2%	18.2% - 24.4%	>24.4%
MI	CRC Resection Rate	<72.9%	72.9% - 72.9%	>72.9%
MI	CRLM Resection rate	<26.1%	26.1% - 26.1%	>26.1%
NJ	CRC Resection Rate	<75.3%	75.3% - 78.6%	>78.6%
NJ	CRLM Resection rate	<24.5%	24.5% - 29.3%	>29.3%
NM	CRC Resection Rate	<61.5%	61.5% - 70.4%	>70.4%
NM	CRLM Resection rate	<6.7%	6.7% - 9.1%	>9.1%
UT	CRC Resection Rate	<79.3%	79.3% - 79.5%	>79.5%
UT	CRLM Resection rate	<22.8%	22.8% - 25.0%	>25.0%
WA	CRC Resection Rate	<77.2%	77.2% - 79.6%	>79.6%
WA	CRLM Resection rate	<22.3%	22.3% - 30.4%	>30.4%

There were 191 counties included in the distance traveled analysis. Patients residing in these counties had access via land-travel to a nearby NCI-designated cancer center or CoC-accredited cancer program, even if this meant crossing state lines. Three counties in Hawaii were excluded due to the need for airflights to visit an NCI-designated cancer center or a CoC-accredited cancer program. Patients living in counties that were concordant and low for both CRC and CRLM surgery had the highest median distance to the nearest NCI-designated cancer center or CoC-accredited cancer program (114.5 and 22.6 miles, respectively, Table 2).

**Table 2:** Colorectal cancer (CRC) and colorectal liver metastasis (CRLM) rates by distance to the closest National Cancer Institute (NCI) and Commission on Cancer (CoC) hospitals* *Cut-off values for high vs. low CRLM and CRC rates: 23.9% and 76.5%, respectively; based on the median. Data represented as median and interquartile range (25th and 75th percentile).

	High CRC, High CRLM: Concordant and High (N=49)	High CRC, Low CRLM: Discordant and Favoring CRC Surgery (N=46)	Low CRC, High CRLM: Discordant and Favoring CRLM Surgery (N=46)	Low CRC, Low CRLM: Concordant and Low (N=50)
Distance from the center of the county to the closest NCI hospital, Median (Q1 - Q3)	63.1 (50.8 to 102.0)	77.1 (44.3 to 137.0)	46.5 (25.8 to 135.5)	114.5 (53.5 to 218.3)
Distance from the center of the county to the closest CoC hospital, Median (Q1 - Q3)	11.3 (6.0 to 26.4)	13.8 (7.0 to 25.6)	11.6 (6.9 to 22.7)	22.6 (9.1 to 40.4)

**Figure 3:**
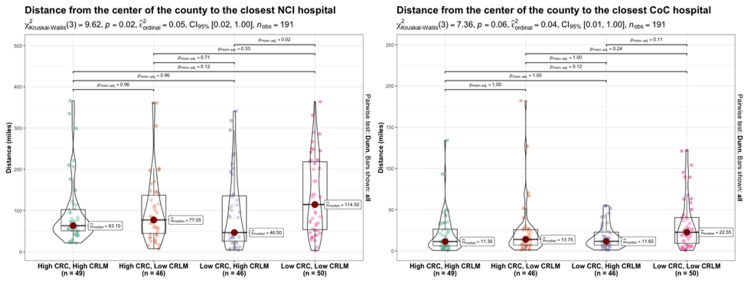
Between-group comparisons for distance and colorectal liver metastasis (CRLM) and colorectal cancer (CRC) rates The Kruskal-Wallis test was used to compare the distribution of counties according to the distance to the closest National Cancer Institute (NCI) and Commission on Cancer (CoC) hospital among the four groups. A sequentially rejective multiple test procedure was used to adjust for multiple comparisons.

Patients living in counties that were discordant (high for CRC surgery and low for CRLM surgery) had the second highest median distance to the nearest NCI-designated cancer center or CoC-accredited cancer program (77.1 and 13.8 miles, respectively). Counties that had high rates of surgery for CRLM had the shortest distance to the nearest NCI-designated cancer center (63.1 and 46.5 miles, respectively). The distance to the nearest NCI-designated cancer center differed significantly between counties that were discordant and favored CRLM surgery and counties that were concordant and low for both CRC and CRLM surgery (p=0.02, Figure 3).

The median distance to the nearest NCI-designated cancer center ranged from 46.5 miles to 114.5 miles among these four groups of categorized counties. Including the 25th and 75th percentiles, these traveling distances ranged from 25.8 miles to 218.3 miles.

## Discussion

Counties with high rates of surgery for CRLM had the shortest distances to the nearest NCI-designated cancer center. This might imply that patients residing in counties with a nearby NCI-designated cancer center might have a greater likelihood of undergoing a liver metastasectomy, which is a complex operation that is more likely to be performed at an NCI-designated cancer center rather than at other types of hospitals. The only statistically significant difference in distance to the nearest NCI-designated cancer center was seen between counties that were discordant and favored CRLM surgery and counties that were concordant and low for both CRC and CRLM surgery. Although distance to an NCI or CoC might be important in determining access to surgery for CRLM or stage I CRC, other factors (e.g., patient- or hospital-level factors such as insurance and hospital expertise and available resources) [2-6] might more fully explain why similar patients are more likely to undergo potentially curative surgery for CRLM or stage I CRC in some counties rather than others.

Previous studies have shown that significant variation exists in how patients with CRLM are managed. Differences in how patients with CRLM are managed have been found between general surgeons and specialized hepatobiliary surgeons and among specialized hepatobiliary surgeons [14,15], illustrating that it matters from whom patients with CRLM receive care. Patients with CRLM who traveled a shorter distance to an NCI-designated hospital are most likely better positioned to undergo a more complex cancer surgery, like a liver metastasectomy, because they have nearby family and/or resources, compared to patients who traveled a farther distance for care. Additionally, specialized hepatobiliary surgical oncologists are more likely to be concentrated in larger cancer centers, like NCI-designated cancer centers. Ultimately, it might matter more where patients with complex cancers, like CRLM, seek and receive care compared to patients with less complex cancers.

This study has certain limitations, including not taking into account type of health insurance or lack thereof, mode of transportation, age at diagnosis, or changes in standard of care treatment over time. However, in our previous study, the proportion of uninsured adults at the county level and the proportion of patients aged 65 years or older were not associated with odds of undergoing liver surgical resection for CRLM at the county level [10]. Mode of transportation is not available, but according to the US Census Bureau 5-Year American Community Survery (2018-2022), as cited by Valentine in Car Ownership Statistics 2024 in Forbes Advisor, the majority of US households, 91.7%, had at least one vehicle in 2022 [16]. Additionally, there have not been significant changes to the standard of care in how stage I CRC or CRLM is treated during the time period spanning 2010-2018 that would confound our findings. When calculating the distance between the center of each county and the location of the nearest NCI-designated cancer center or CoC-accredited cancer program, we used Google Maps, and this has not been validated. Last, this is an ecological analysis at the county level, and we do not have any information on where exactly patients sought and/or received care. The ecological nature of the study limits causal inference.

Going beyond differences in traveling distance between these groups of counties, these traveling distances to the nearest NCI-designated cancer center are potentially prohibitive for many patients. Some patients are potentially travelling up to 100-200 miles to their nearest NCI-designated cancer center. Ultimately, the decision to proceed with curative-intent surgery involves more than distance to a facility and this study does not consider logistical, psychological, or patient-specific medical considerations. Further research, preferably with more granular data, will be crucial in validating our observations. Ultimately, potential solutions need to take into account how far patients need to travel for care, and should focus on care integration across multiple institutions that span multiple counties in order to ensure all patients are being appropriately evaluated and receiving the recommended cancer care.

## Conclusions

At the county level, there is variation in undergoing curative surgery for CRLM or stage I CRC. Patients residing in counties with high rates of surgery for CRLM had the shortest median distance to the nearest NCI-designated cancer center. Although median traveling distances to the nearest NCI-designated cancer center were potentially prohibitive, they did not fully explain differences in county-level surgical rates for CRLM. Other factors like logistical, psychological, or patient-specific medical considerations might play a role in explaining county-level differences in surgical rates for CRLM and stage I CRC.
